# Cross-border dual-channel supply chain decision-making under random demand and tariff conditions

**DOI:** 10.1371/journal.pone.0297923

**Published:** 2024-02-12

**Authors:** Fuchang Li, Zhongwei Yang, Xiaohui Hu

**Affiliations:** School of Economics and Management, Yunnan Normal University, Kunming, China; Hosei University: Hosei Daigaku, JAPAN

## Abstract

Given the cross-border e-commerce import tariff and random demands, this study establishes a pricing decision model for cross-border e-commerce dual-channel supply chain, which is composed of domestic manufacturers and overseas retailers, so as to analyze the effects of import tariff and random demand on the pricing, demand and profit of cross-border e-commerce. According to the research, import tariffs have a positive correlation with retailers’ retail prices and a negative correlation with manufacturers’ direct prices, wholesale prices, demand and profit from direct channels, and profit from retail channels. The export tax rebate policy will lessen the negative effects of import tariffs and maximize the best choices made by manufacturers and retailers.

## 1. Introduction

Cross-border e-commerce has significantly changed the global trade landscape. The convenience of internet shopping combined with changing consumer tastes may be seen in the growing popularity of purchasing foreign goods through e-commerce platforms This shift in consumption patterns has motivate manufacturers to modify their distribution strategies. Though many manufacturers are currently aggressively investigating cross-border e-commerce platforms to directly access customers, traditional distribution channels remain relevant. Online platform enterprises have also expanded their cross-border e-commerce business to help local manufacturers set up online sales channels. The industrial sector has benefited from this trend, as seen by the rise in cross-border e-commerce import and export from China.

Trade across borders is not without difficulties. The rivalry between the two channels- traditional distribution channels and international e-commerce- is one of the problems. In order to successfully traverse this complex market, manufacturers should carefully assess their pricing and sales tactics. There are numerous things to think about, including: More overseas customers are now able to learn about and decide to buy items from domestic producers thanks to the development of cross-border e-commerce platforms. As a result, there is increased unpredictability and unpredictable market demand in the outside market.

Import tariffs can impact the cross-border supply chain by rising costs and potentially reducing the competitiveness of products. Manufacturers should evaluate the effects of tariffs and take them into account when setting their prices and managing their supply chains.

Additionally, the cross-border supply chain may be impacted by the export tax refund policy. Changes in the policy may affect the profitability and competitiveness of exporters. Manufacturers and retailers engaged in international trade ought to remain informed about any modifications to policy and modify their tactics correspondingly.

Cross-border dual-channel strategies are more complicated and take into account a wider range of circumstances than domestic dual-channel strategies. As a result, both domestic producers and foreign merchants must consider how to modify pricing and sales strategies in the context of cross-border supply chains. Numerous academics have carried out pertinent study.

Dual-channel supply chains have received a lot of attention in the past few years. Previous research mostly used game theory to investigate the coordination issue in dual-channel supply chains. A thorough analysis of conflict modeling techniques in dual-channel supply chains was given by Tsay and Agrawal. Next Yao and Liu [1, 2] adopted a game model to exmine pricing competition between two channels. Cattani et al. [3] suggested that online price adjustments are one way that producers might lessen dispute. Further research has broadened and enhanced the pricing decisions of dual-channel supply chains. Chiang et al. [4] studied the pricing game between manufacturers and retailers based on a consumer choice model and discovered that the direct channel boosts the manufacturer’s revenue. Huang et al. [5] looked into the pricing decisions of both the manufacturer and retailer. Chen et al. [6] investigated the effects of pricing and advertising on dual-channel competition. Matsui [7] examined the pricing schedule of manufacturers. Price-based strategies for reducing channel conflict were investigated by Zhang and Wang [8]. As a result, further research has improved the analysis of channel conflict reduction and expanded the range of variables influencing pricing choices. Furthermore, information exchange is a crucial component in fostering dual-channel coordination [9]. Kong et al. [10] proposed that revenue sharing encourages information exchange and lessens information leakage. Lei et al. [11] examined the coordination of dual-channel supply chains from the perspective of information asymmetry. At present, the academic circle has conducted a comprehensive study on the price competition, channel conflict and coordination mechanism of dual-channel supply chain under different game conditions. However, there is a shortage of researches related to specific policies. This research presents import tariff as a specific policy factor and random demand in the setting of cross-border e-commerce dual-channel supply chain. It is a reasonable supplement to the research field.

Initially, when it comes to how tariffs effect cross-border trade, the general consensus among academics is that they negatively disrupt supply chains as importing countries impose tariffs to suppress foreign firms’ exports and protect domestic industries. According to Chen [12], the trade in intermediate items is adversely affected by tariff impositions. From a cost perspective, Tian [13] believed that lowering tariffs could reduce the cost of intermediate goods imported by enterprises and improve the export intensity of enterprises. Yu and Yuan [14] pointed out that while a decrease in domestic end product tariffs may lower an enterprise’s cost markup, a fall in overseas tariffs and input tariffs will raise it. Wang [15] comes to the conclusion that imposing tariffs on importing nations raises the cost of trading in intermediate goods for domestic businesses, which raises the cost of exporting and makes it less advantageous for home businesses to export. Rong and Xu [16] demonstrate that higher tariffs harm the interests of manufacturers and retailers and impede the growth of international trade to some extent by restricting the development of cross-border green supply chains. According to Maria and Luca’s [17] EU survey, reduced tariffs on agricultural products directly increase the number of imports. At the same time, tariffs will not only affect the cost of cross-border business but also affect other aspects of cross-border supply chains. According to Niu et al. [18], overseas sourcing not only entails high wholesale prices and tariff costs but also make people reliant on foreign sources for technology. As Liu [19] shows, tariffs imposed by importing nations can have a cascading effect both upstream and downstream, which raises domestic prices and jeopardizes consumer welfare in addition to decreasing the marginal profit of exported goods and stifling market demand. Meanwhile, Tian and Yu [20] discover that lower R&D overheads result from lower intermediate tariffs, which boosts businesses’ ability to innovate. Liu and Qiu [21], on the other hand, contend that lowering tariffs on intermediate goods actually lowers the price of premium imported inputs and encourages the replacement of domestic inventions. Nonetheless, some academics have also contended that, depending on the circumstances of the supply chain, different tariffs may have particular advantages.

According to Hsu and Zhu [22], a strategy of reallocation at the final product level may be made possible by a decrease in the tax rebate rate with regard to export tax refunds. Meanwhile, there is a threshold value for the VAT refund rate in reasonable and moderate business conditions. Liu et al. [23] adjusted the newsboy model for two distinct models (centralized and decentralized) to investigate Chinese companies’ supply chain choices in light of the country’s export-oriented tax and value-added tax (VAT) policies. It is discovered that the export tax rebate policy has an impact on the ideal order quantity and profit distribution under both models. According to Xu et al. [24], raising the VAT refund rate will benefit foreign businesses while hurting domestic ones. A quantitative competition model is created by Nagurney [25] to investigate how export performance is affected by export tax rebate policy. The conclusion implies that tariff quotas benefit home producers while potentially raising prices for consumers. Using China as an example, Braakman et al. [26] investigate the effects of changing export tax rebate rates on export pricing. The study demonstrates that negative adjustments to VAT refunds result in considerable drops in export value and volume, respectively. The body of research indicates that trade flows, firm operations, and profitability are significantly impacted by export tax rebate policies. Additional investigation may be conducted to determine the best tax refund plan and how it interacts with other trade regulations.

Examining the impact of tariffs on cross-border supply chain coordination and decision-making has been a major area of academic interest. Cole and Eckel [27] discovered that merchants’ markup effects have an impact on how tariffs are passed up the supply chain to other commodities. According to Duan [28], the value chain effect of tariffs will cause a decline in the effective protection rate of tariffs for downstream sectors or nations, but an increase in tariffs will strengthen the protection of local industries. Liang and Liang [29] investigated the effects of changes in domestic import duties on pricing choices and profitability in supply chains with two channels, providing further insight into the effects of tariffs. Coordination strategies have been proposed by academics to optimize networks that are subject to swings in exchange rates and tariffs. Yue and Zhao [30] analyzed the impact of exchange rate and tariff changes on cross-border supply chains and proposed quantity discount contracts to coordinate the supply chain. Zhao [31] investigated the coordination issue of cross-border supply chains under the combination contract of transfer payments and quantity discounts in order to further analyze coordination. Supply chain models have also evaluated the effects of tariff policies on trade flows and operations. In order to investigate the effects of cross-border variables like tariff deficiencies and transportation costs on cross-border supply networks, Villegas and Ouenniche [32] built a model of production and distribution planning for cross-border supply chains. International manufacturers, domestic suppliers, and third-party integrated international logistics service providers make up the supply chain decision-making model that Hu et al. [33] built. The study demonstrates how the imposition of tariffs has seriously disrupted the functioning of global trade and supply chains. Generally speaking, research underscores how tariffs significantly affect cross-border coordination, necessitating mechanisms, like contracts, to optimize decision-making. Further studies could assess emerging coordination approaches and dynamic optimization under different policies.

The influence of export rebates and import tariffs on cross-border e-commerce supply chains is well-documented in the research now under publication [34–36]. Few research on pricing and coordination under the complicated tax system fully take into account the dual-channel cross-border supply chain model, erratic demand, and particular regulations. In order to provide insights for real-time decision optimization under tariffs, export tax rebates, and random demand variations, future research should build creative coordination methods for dual-channel supply chains in the context of import tariffs and export tax rebates.

Therefore, gaining a deeper understanding of the impact mechanism of tariffs on cross-border e-commerce supply chains can be achieved by looking at the effect of tariffs from the perspective of cross-border e-commerce supply chain operation and by revealing the micro mechanism of tariffs affecting pricing and inventory decisions of enterprises through transmission along the supply chain. This paper’s realistic background and well-defined content can enrich the research on pricing and coordination of cross-border dual-channel supply chain under complex tax system. Various factors have been introduced into cross-border dual channel supply chain model, including import tariff, export tax rebate and random demand. Then, the two-stage Stackelberg game decentralized decision model is established to solve and analyze the influences of the tariff, export tax rebates and random demand on the supply chain optimal pricing, demand and profit. Lastly, numerical example is given to verify the rationality of the model in real world.

## 2. Basic assumptions and model descriptions

Based on the differential pricing, a dual-channel supply chain system with a secondary level of cross-border e-commerce is built: In this system, there are three parties, including a domestic manufacturer, an overseas retailer, and overseas consumers. The product is produced domestically by the manufacturer, and the unit cost is denoted as *c*, a direct price at which the manufacturer sells its goods to customers abroad, denoted as *p*_*r*_. Furthermore, there is a retail price that the retailer charges foreign consumers, denoted as *w*, and a retail price at which the retailer sells the product to consumers abroad, denoted as *p*_*r*_. *p*_*m*_ *≠ p*_*r*_.

Hypothesis 1: Foreign retailers import unit products and need to pay customs duties. The tariff ratio is *t*, and retailers often transfer the tariff cost to consumers through tax-inclusive prices. that is, the final retail price of products includes customs duties.
Hypothesis 2: In addition to paying the direct sales price, overseas consumers need to pay customs duties when purchasing Chinese products on cross-border e-commerce platforms. The tariff ratio is *t*.
Hypothesis 3: In order to facilitate calculation, manufacturers and retailers do not consider other costs, and overseas consumers do not have to bear additional costs for purchasing products.
Hypothesis 4: The government provides the export tax rebate policy to the domestic manufacturing industry. The export tax rebate is the production cost multiplied by the export tax rebate rate, in which the export tax rebate rate is *d*.

Stochastic demand perturbation combined with the linear demand model is a popular treatment approach in operations research and management science. This approach preserves the computational simplicity and solvable model while somewhat reflecting the unpredictability of demand. Numerous operations research and management science journals accept and employ this approach extensively. It makes sense to use a simplified linear demand model as the goal of this research is to investigate the circumstances in a particular economic environment. In subsequent research, a full stochastic uncertainty demand model may be investigated. Under random demand, *D*_*m*_ and *D*_*r*_ represent the demand for manufacturers’ cross-border e-commerce direct sales channels and the demand for retailers’ traditional retail channels, respectively. *ρ* is the consumer′s preference coefficient for traditional retail channels (*ρ* ≥ 0), (1− *ρ*) is the consumer’s preference coefficient for cross–border e–commerce direct sales channels, *a* is the total demand of overseas dual-channel markets, *b* is the consumer′s sensitivity to the price of the product (*b* > 0), and *γ* is the elasticity of demand between channels (*b* > *γ* > 0). Due to the uncertainty of the foreign market, there is a random demand in the market. The uncertain demand of the cross-border e-commerce direct sales channels, denoted as *ε*_*m*_. Additionally, it is that *ε*_*m*_ follows a random distribution with an expected value of *μ*_*m*_ and a variance of $\sigma_{m}^{2}$ on (*m*_*m*_, *n*_*m*_). The uncertain demand of the traditional retail channel, denoted as *ε*_*r*_. Additionally, it is that *ε*_*r*_ follows a random distribution with an expected value of *μ*_*r*_ and a variance of $\sigma_{r}^{2}$ on (*m*_*r*_, *n*_*r*_). They reflect the randomness of market demand for the two channels. Then, the manufacturer and retailer expect the demand function *D*_*m*_, *D*_*r*_ respectively:

$$D_{m}=\rho \alpha -b(1+t)p_{m}+\gamma p_{r\;}+\mu_{m}$$ (1)

$$D_{r}=\left(1-\rho \right)\alpha -bp_{r\;}+\gamma (1+t)p_{m}+\mu_{r}$$ (2)

The decision objective functions for manufacturers and retailers are shown as follows:

$$\pi_{m}=[p_{m}-(1-d)c]D_{m}+[w-(1-d)c]D_{r}$$ (3)

$$\pi_{r}=[p_{r\;}-(1+t)w]D_{r}$$ (4)

## 3. Model solution and result analysis

### 3.1 Model solving

A domestic producer and an international store are involved in the supply chain; both engage in the Stackelberg game. With the intention of maximizing their own profits, domestic manufacturers determine the wholesale price of goods and the sales price of cross-border e-commerce direct sales channels in the first stage. In the sales stage, zero foreign sellers determine the sales price of traditional retail channels.

**Proposition 1**: When *b* > *γ*, the best decision for manufacturers and retailers is:

$${p_{m}}^{*}=\frac{a\gamma +\gamma \mu_{r}+b\mu_{m}+c(b^{2}-\gamma^{2})(1+t)(1-d)+a\rho (b-\gamma )}{2(b^{2}-\gamma^{2})(1+t)}$$ (5)

$$w^{*}=\frac{ab+b\mu_{r}+\gamma \mu_{m}+c(b^{2}-\gamma^{2})(1+t)(1-d)-a\rho (b-\gamma )}{2(b^{2}-\gamma^{2})(1+t)}$$ (6)

$${p_{r}}^{*}=\frac{2(b^{2}-\gamma^{2})[\left(1-\rho \right)\alpha +\mu_{r}+c(1+t)(1-d)]+(a+\mu_{r}+\mu_{m})(b+\gamma )}{4b(b^{2}-\gamma^{2})}$$ (7)

Proof: The reverse induction method is used to solve the problem. Firstly, according to Eq (4), the retailer’s first-order guide is obtained: $\frac{\partial \pi_{r}}{\partial p_{r}}=-2bp_{r\;}+\left(1-\rho \right)\alpha +\gamma \left(1+t\right)p_{m}+\mu_{r}+\left(1+t\right)w$. Because $\frac{\partial^{2}\pi_{r}}{\partial {p_{r}}^{2}}=-2b<0,\;\pi_{r}$ is about the concave function of $p_{r\;}.\;\frac{\partial \pi_{r}}{\partial p_{r}}=0$, then $p_{r\;}=\frac{\left(1-\rho \right)\alpha +\gamma \left(1+t\right)p_{m}+\mu_{r}+\left(1+t\right)w}{2b}$. Substitute *p*_*r*_ into Eq (3) and simplification. Then obtain the first derivatives *w* and *p*_*m*_ for *π*_*m*_, that is $\frac{\partial \pi_{m}}{\partial w},\;\frac{\partial \pi_{m}}{\partial p_{m}}$, respectively. Then, the second derivative of *w* and *p*_*m*_ is obtained to get the Hese matrix $H=\left[\begin{array}{cc} \frac{\left(\gamma^{2}-2b^{2}\right)\left(1+t\right)}{b} & \gamma \left(1+t\right)\\ \gamma \left(1+t\right) & -b\left(1+t\right) \end{array}\right]$. Thus, 2*b*^2^ − 2*γ*^2^ > 0, namely b>γ. The Heiser matrix H is negatively definite. That is, the manufacturer’s profit *π*_*m*_ is a concave function of *w and p*_*m*_. $\frac{\partial \pi_{m}}{\partial w}$ and $\frac{\partial \pi_{m}}{\partial p_{m}}$ equal to 0, so that w* *and p*^m^* can be obtained. Then w*, *p*_m_* can be substituted into *p*_*r*_. After simplifying, *p*_r_* is obtained. Proposition 1 is proven.

From proposition 1, the optimal market demand of traditional retail channels, the optimal market demand of cross-border e-commerce network direct sales channels, the optimal profit of manufacturers and the optimal profit of retailers are obtained respectively:

$${D_{m}}^{*}=\rho \alpha -b(1+t){p_{m}}^{*}+\gamma {p_{r}}^{*}+\mu_{m}$$ (8)

$${D_{r}}^{*}=\left(1-\rho \right)\alpha -b{p_{r}}^{*}+\gamma (1+t){p_{m}}^{*}+\mu_{r}$$ (9)

$${\pi_{m}}^{*}=[{p_{m}}^{*}-(1-d)c]{D_{m}}^{*}+(w^{*}-[1-d)c]{D_{r}}^{*}$$ (10)

$${\pi_{r}}^{*}=[{p_{r}}^{*}-(1-t)w^{*}]{D_{r}}^{*}$$ (11)

### 3.2 Analysis of results

**Corollary 1**: In cross-border dual-channel supply chains, there is: 1) $\frac{\partial p_{m}*}{\partial t}<0,\;\frac{\partial w^{*}}{\partial t}<0,\;\frac{\partial p_{r}*}{\partial t}>0$; 2) $\frac{\partial D_{m}*}{\partial t}<0,\;\frac{\partial D_{r}*}{\partial t}<0$.

Corollary 1 proves that:

1) Increase in import tariff percentage negatively affects the optimal decision of the supply chain.

The optimal decision for domestic manufacturers’ products’ direct sales prices and wholesale prices of cross-border e-commerce channels declines as the share of import duties rises, while the retail prices of foreign merchants rise. Manufacturers lower their direct sales prices and wholesale prices to ensure sales, and foreign retailers raise their retail prices to ensure profits. These changes in import tariffs have an impact on foreign retailers’ and consumers’ actual purchase prices, meaning that their purchase costs rise. Generally speaking, across the supply chain, optimal decision-making prices are negatively impacted by changes in import duties.

2) Increase in import tariff percentage reduces demand for dual channels

As the proportion of import tariffs increases, the demand for cross-border e-commerce channels and the demand for traditional retail channels will decrease. The increase in import tariffs will increase the purchase cost of foreign retailers and consumers, which will affect the direct sales price and retail price, which in turn reduces the demand for direct sales channels and retail channels.

Proof: Derived analysis can be proved.

**Corollary 2**: In – cross – border dual – channel supply chains, there is: $\frac{\partial {\pi_{m}}^{*}}{\partial t}<0,\;\frac{\partial {\pi_{r}}^{*}}{\partial t}<0$.

Corollary 2 shows that import tariffs reduces profits on both sides of the supply chain.

The manufacturer’s profit and the retailer’s profit both decline as import tariffs rise. Increased import tariffs will have a negative effect on domestic manufacturers by lowering direct sales prices as well as the demand for retail and direct sales channels, which will significantly diminish manufacturers’ earnings. Increased import levies will have the effect of making orders more expensive for overseas retailers and decreasing demand for retail channels, which will lower retailer earnings. This implies that import duties hurt imports and exports by squeezing the earnings of producers and merchants. Proof: Derived analysis can be proved.

**Corollary 3**: In cross-border dual-channel supply chains, there is: 1) $\frac{\partial p_{m}*}{\partial d}<0,\;\frac{\partial w^{*}}{\partial d}<0,\;\frac{\partial p_{r}*}{\partial d}<0$; 2) $\frac{\partial D_{m}*}{\partial d}>0,\;\frac{\partial D_{r}*}{\partial d}>0$.

Corollary 3 shows that:

1) An increase in the export tax rebate rate positively affects the optimal decision of the supply chain.

With the increase of export tax rebate ratio, the direct selling price of cross-border e-commerce channels, the optimal decision of wholesale price and the retail price of overseas retailers will decrease. The increase of export tax rebate rate will affect the production cost of manufacturers. In order to increase the sales volume, manufacturers will choose to reduce the direct selling prices and wholesale prices. Therefore, the purchase cost of foreign retailers will also decrease, thus reducing the retail price.

2) Increase in export tax rebate rate increases dual-channel demand

With the increase of export tax rebate proportion, the demand for cross-border e-commerce channels and the demand of traditional retail channels will decrease. The increase of export tax rebate will reduce the purchase cost of foreign retailers and consumers, and will affect the direct selling price and retail price, and then increase the demand of direct sale channel and retail sale channel.

Proof: Derived analysis can be proved.

**Corollary 4**: In cross-border dual-channel supply chains, there is: $\frac{\partial {\pi_{m}}^{*}}{\partial d}>0,\;\frac{\partial {\pi_{r}}^{*}}{\partial d}>0$.

Corollary 4 shows that export tax rebates increase the profits of both sides of the supply chain. As export tax rebates increase, not only will manufacturers ’profits increase, but also retailers’ profits will increase. Because with the increase of export tax rebate rate, the manufacturer’s cost will decrease, the price of goods will decrease, and the demand of consumers will increase because of the decreasing price, thus the manufacturer’s profit will increase. Similarly, with the increase of export tax rebate rate, the import cost of the seller will decrease, the price of goods will decrease, and the demand of consumers will increase because of the decrease of the price, increasing the profit of the retailer.

Proof: Derived analysis can be proved.

**Corollary 5**: In cross-border dual-channel supply chains, there is: 1) $\frac{\partial p_{m}*}{\partial \mu_{m}}>0,\;\frac{\partial w^{*}}{\partial \mu_{m}}>0,\;\frac{\partial p_{r}*}{\partial \mu_{m}}>0;\frac{\partial p_{m}*}{\partial \mu_{r}}>0,\;\frac{\partial w^{*}}{\partial \mu_{r}}>0,\;\frac{\partial p_{r}*}{\partial \mu_{r}}>0$; 2) $\frac{\partial D_{m}*}{\partial \mu_{m}}>0,\;\frac{\partial D_{r}*}{\partial \mu_{m}}=0,\;\frac{\partial D_{m}*}{\partial \mu_{r}}>0,\;\frac{\partial D_{r}*}{\partial \mu_{r}}>0$.

Corollary 5 shows that:

1) Increased random demand increases dual-channel profits

The direct sales price, wholesale price, and retail price of products from foreign merchants will rise in response to the growth in random demand, whether it comes from direct sales channels or retail channels. In order to maximize profits, producers will raise direct sales prices and wholesale prices, and overseas retailers will likewise raise retail prices in response to the increase in random demand. These actions will have an impact on the real sales volume of manufacturers and retailers.

2) Dual channels increase prices while still increasing overall demand

Traditional retail channels will continue to be in demand, but the demand for cross-border e-commerce channels will rise in tandem with the erratic demand for direct sales channels. The need for cross-border e-commerce and traditional retail channels will rise in tandem with the increase in sporadic demand for retail channels. The demand for traditional retail channels and cross-border e-commerce channels has increased overall, despite a decline in demand due to rising direct selling and retail costs. Proof: Derived analysis can be proved.

**Corollary 6**: In cross-border dual-channel supply chains, there is: $\frac{\partial {\pi_{m}}^{*}}{{\partial \mu }_{m}}>0,\;\frac{\partial {\pi_{r}}^{*}}{{\partial \mu }_{m}}=0;\frac{\partial {\pi_{m}}^{*}}{{\partial \mu }_{r}}>0,\;\frac{\partial {\pi_{r}}^{*}}{{\partial \mu }_{r}}>0$.

Corollary 6 suggests that changes in stochastic demand show different effects across channels. Manufacturer earnings rise in response to random demand from direct sales channels; retailer profits are unaffected. Manufacturers and retailers experience a rise in earnings in tandem with an increase in random demand from retail channels. The profit margins of manufacturers and retailers rise in tandem with an increase in pricing, random demand, direct channel demand, and retail channel demand. Proof: Derived analysis can be proved.

## 4. Numerical examples

The implications of import tariffs, export tax rebates, and random demand on the optimum pricing, demand, and profits for supply chain participants in dual-channel supply chains are covered in this section.

### 4.1 The impact of import tariffs on supply chain decision-making

This subsection discusses the effect of changes in import tariffs and export tax rebates on the optimal decisions of domestic manufacturers and overseas retailers. To study the effects of import tariffs and export tax rebates in the context of cross-border e-commerce, this study carries out numerical simulation study using actual data from a business that sells its goods to customers abroad via cross-border retailers and online marketplaces. To protect privacy, this paper refers to the company as "NC." NC’s product line mainly includes neck cold compresses. The main data collection methods include offline survey and online analysis:

1) Offline research: Sales representatives of "NC Company" were interviewed to learn more about their dual-channel pricing strategy, cross-border retail environment, and how import tariffs and export tax rebates affect their business.
2) Online analysis: A thorough analysis of NC’s neck cold compress, the company’s flagship product, has been conducted by the online sales platform. This entails being aware of the product’s pricing, costs, and online market demand.

NC’s raw data are standardized for easier understanding and comparison. In order to more effectively express the data, it must be scaled to a common scale without distorting range differences or removing information. Based on the survey and processing steps mentioned above, the relevant data are as follows: *α* = 100, *b* = 1.5, *γ* = 0.7, *ρ* = 0.6, *c* = 20, *μ*_*m*_ = 5, *μ*_*r*_ = 6, *t* ∈ [0, 0.5], *d* ∈ [0, 0.2].

#### 4.1.1 Effect of import tariff and export tax rebates on the optimal decision price in the supply chain

**Fig 1:**
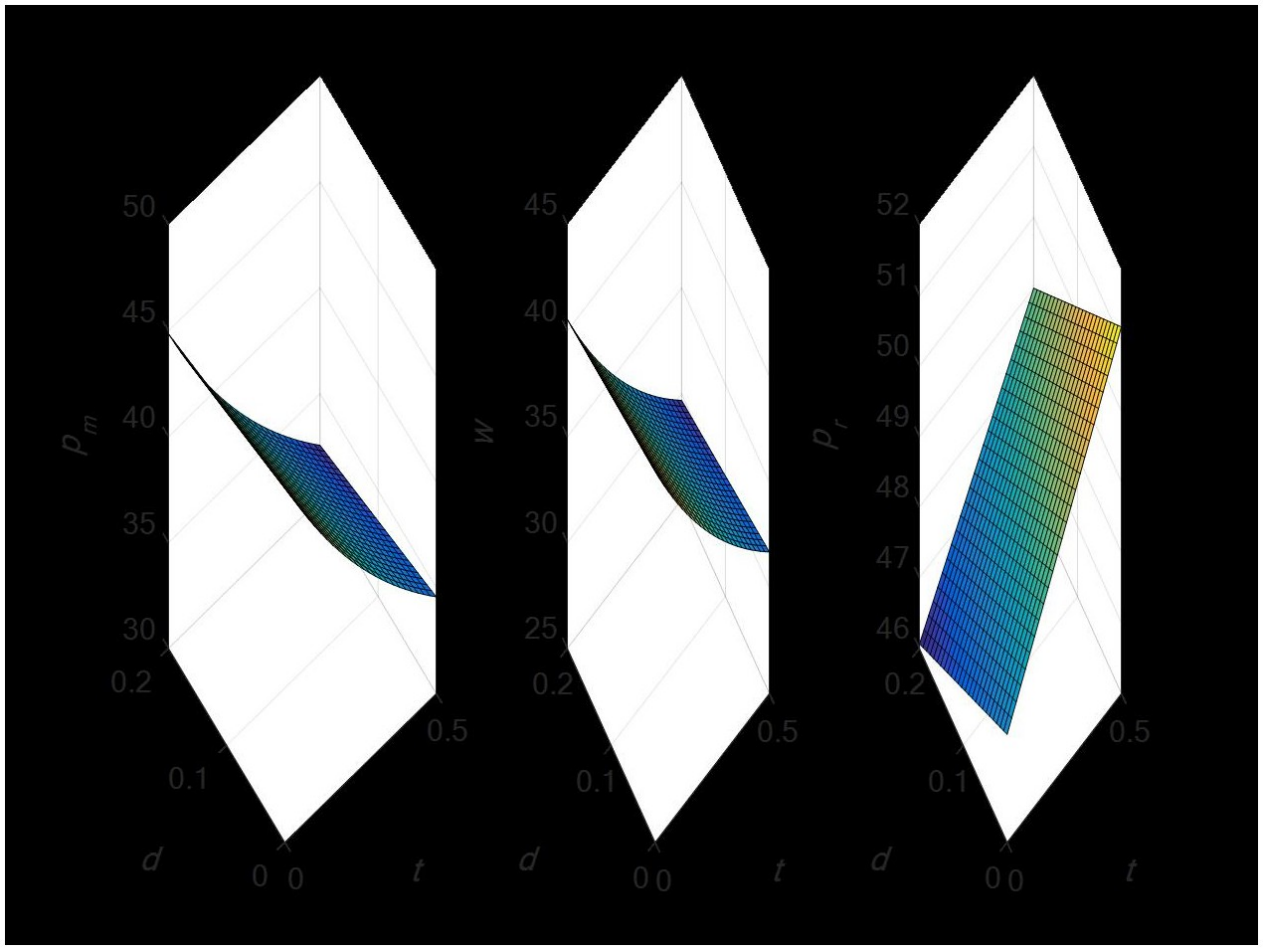
Effect of tariff changes and export tax rebate on the optimal decision price.

Fig 1 illustrates the negative link between the import tariff rate and wholesale and direct selling prices, and the positive association it has with retail pricing. This suggests that adjustments to import tariffs may have an impact on supply chain pricing. Conversely, there is an inverse link between the export tax refund rate and wholesale, retail, and direct selling prices. Retail prices would rise in an instance where there was a simultaneous increase in the import tariff and the export tax rebate rate, while direct selling and wholesale prices would decline.

#### 4.1.2 The impact of import duties and export tax rebates on the demand of the dual-channel supply chain

**Fig 2:**
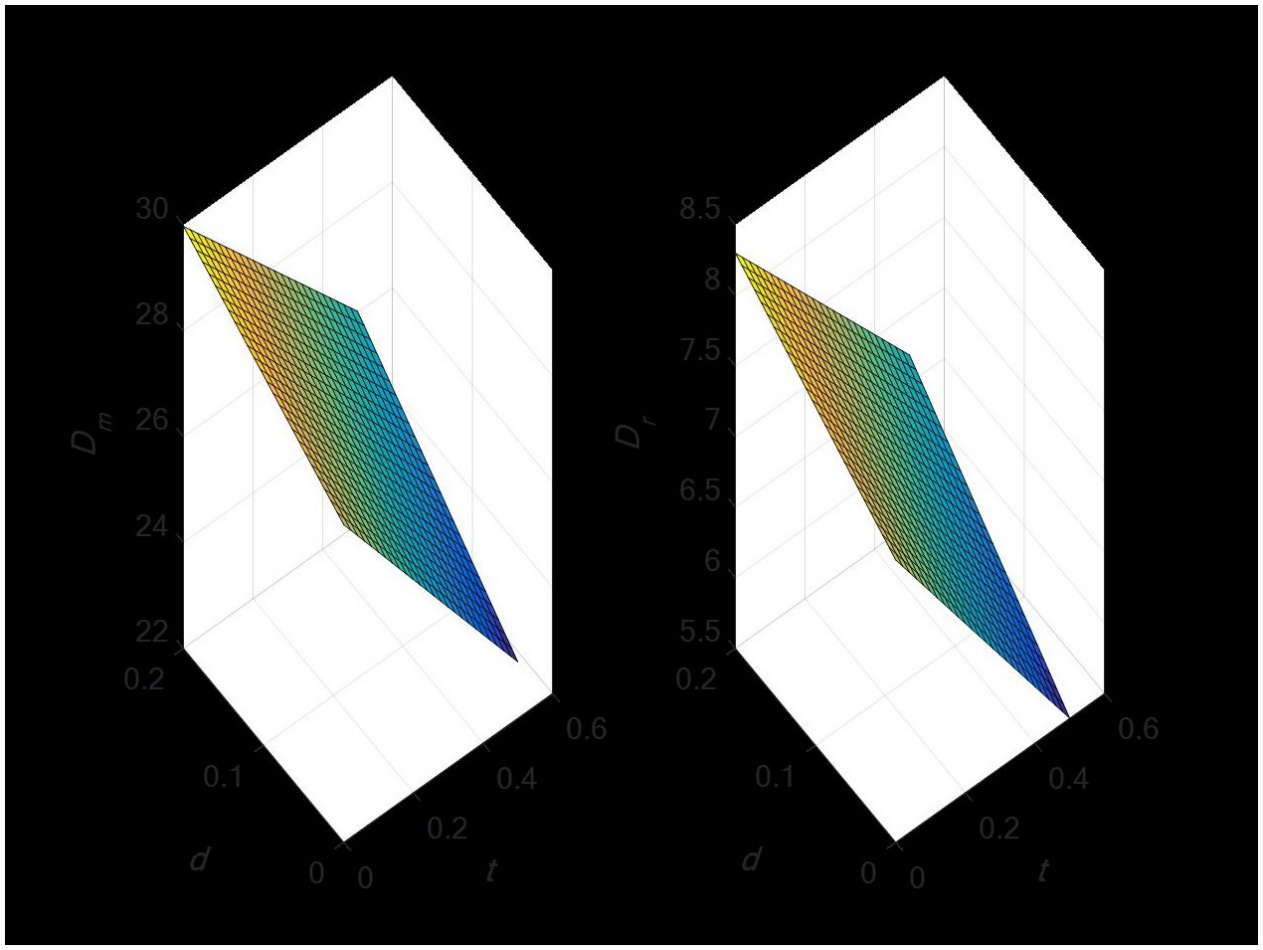
Impact of tariff changes and export tax rebate on dual-channel demand.

According to Fig 2, the import tariff rate has an inverse relationship with demand in the direct sales and the retail channels. This indicates that changes in import tariffs can affect demand in the supply chain. Also, the export tax rebate rate shows a positive relationship with demand in the direct sales and the retail channels. This suggests that the export tax rebate rate can influence demand in the supply chain. Demand in the retail and direct sales channels decline when both the import tariff and the export tax rebate rate increase. The export tax rebate policy, however, has the potential to somewhat mitigate the impact of import tariffs on demand.

#### 4.1.3 The impact of import tariff and export tax rebate on the profit of the dual-channel supply chain

**Fig 3:**
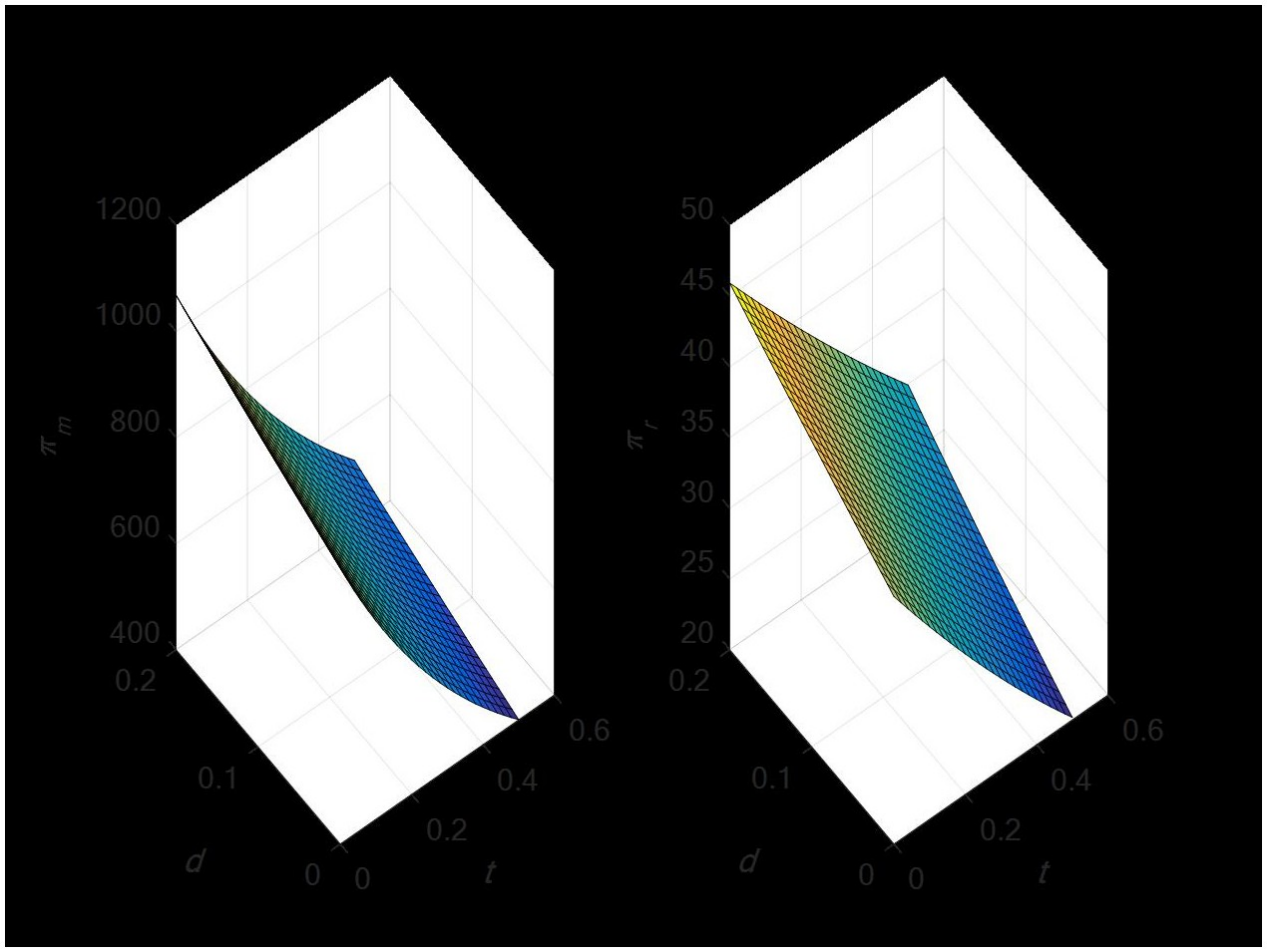
Impact of tariff change and export tax rebate on the profit of the dual-channel supply chain.

Fig 3 illustrates the inverse link between the import tariff rate and store and producer earnings. This suggests that adjustments to import tariffs may have a substantial negative impact on supply chain earnings. Ultimately, producers’ and retailers’ best options will be somewhat impacted by higher tariffs on imports, and both effects are negative. Additionally, consumers will pay more for their purchases. Furthermore, domestic producers are more affected by increases in import tariffs than are foreign retailers. As a result, import tariffs will lower both imports and global trade. Conversely, there is an inverse link between manufacturer and store earnings and the export tax refund rate. This implies that supply chain profitability may be impacted by the export tax refund rate.

Demand in the direct sales channel declines along with retail channel earnings when both the import tariff and the export tax rebate rate rise. However, to some extent, the export tax rebate policy can lessen the adverse impact of import tariffs on profits.

### 4.2 The impact of random demand on supply chain decision-making

This subsection discusses the impact of changed import tariff on the optimal decisions of domestic manufacturers and overseas retailers. The relevant parameters are set as the following: *α* = 100, *b* = 1.5, *γ* = 0.7, *ρ* = 0.6, *c* = 20, *t* = 0.1, *d* = 0.13, *μ*_*m*_ ∈ [0, 5], *μ*_*r*_ ∈ [0, 5].

#### 4.2.1 The influence of random demand on the optimal decision-making price of the supply chain

**Fig 4:**
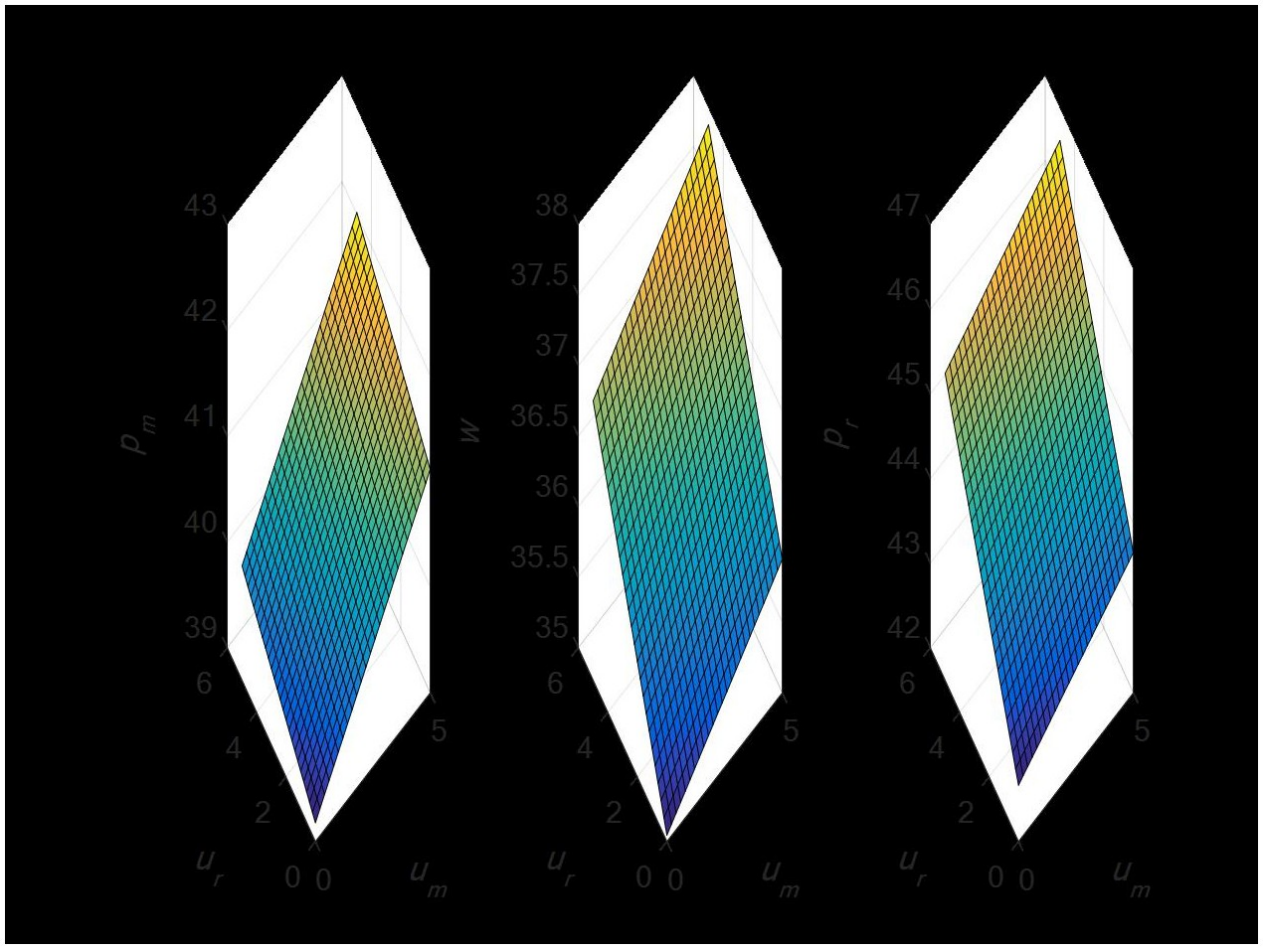
The influence of random demand on the optimal decision price.

According to Fig 4, the retailer’s retail price and the manufacturer’s direct sales price are both positively impacted by the random demand of the direct sales channel, and both have a positive change relationship. Similarly, random demand in retail channels affects retail prices as well as direct sales and retail prices. Thus, manufacturers and retailers set higher selling prices based on random demand.

#### 4.2.2 The impact of random demand on the demand of dual-channel supply chain

**Fig 5:**
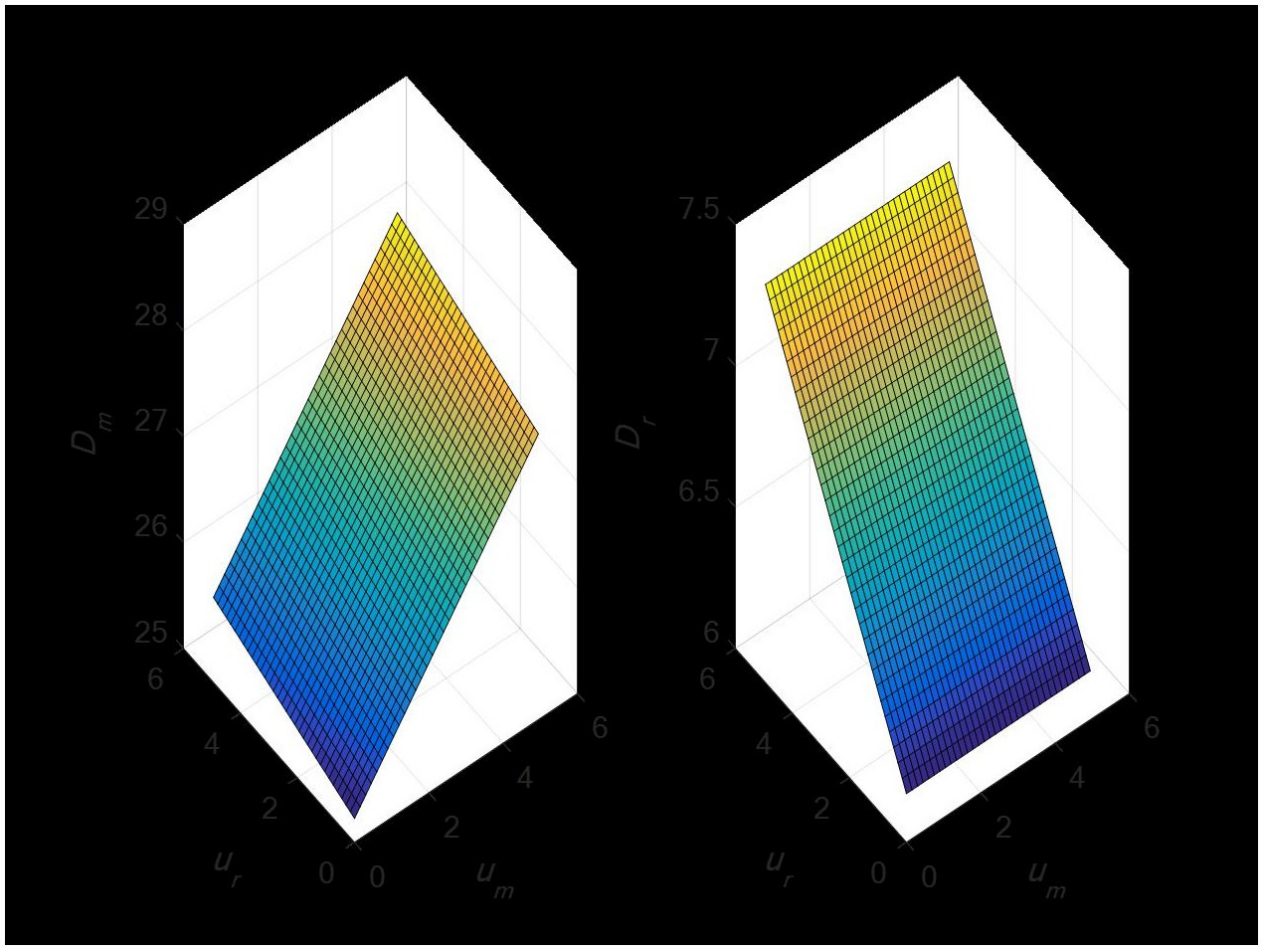
The impact of random demand on dual-channel demand.

The demand of retail channels will not be impacted by the erratic demand of direct sales channels, as Fig 5 demonstrates. Instead, it only has a positive change relationship with the demand of direct sales channels. Nonetheless, sporadic demand for retail channels will have a favorable impact on both direct sales and retail channel demand.

#### 4.2.3 The impact of random demand on the profit of dual-channel supply chain

**Fig 6:**
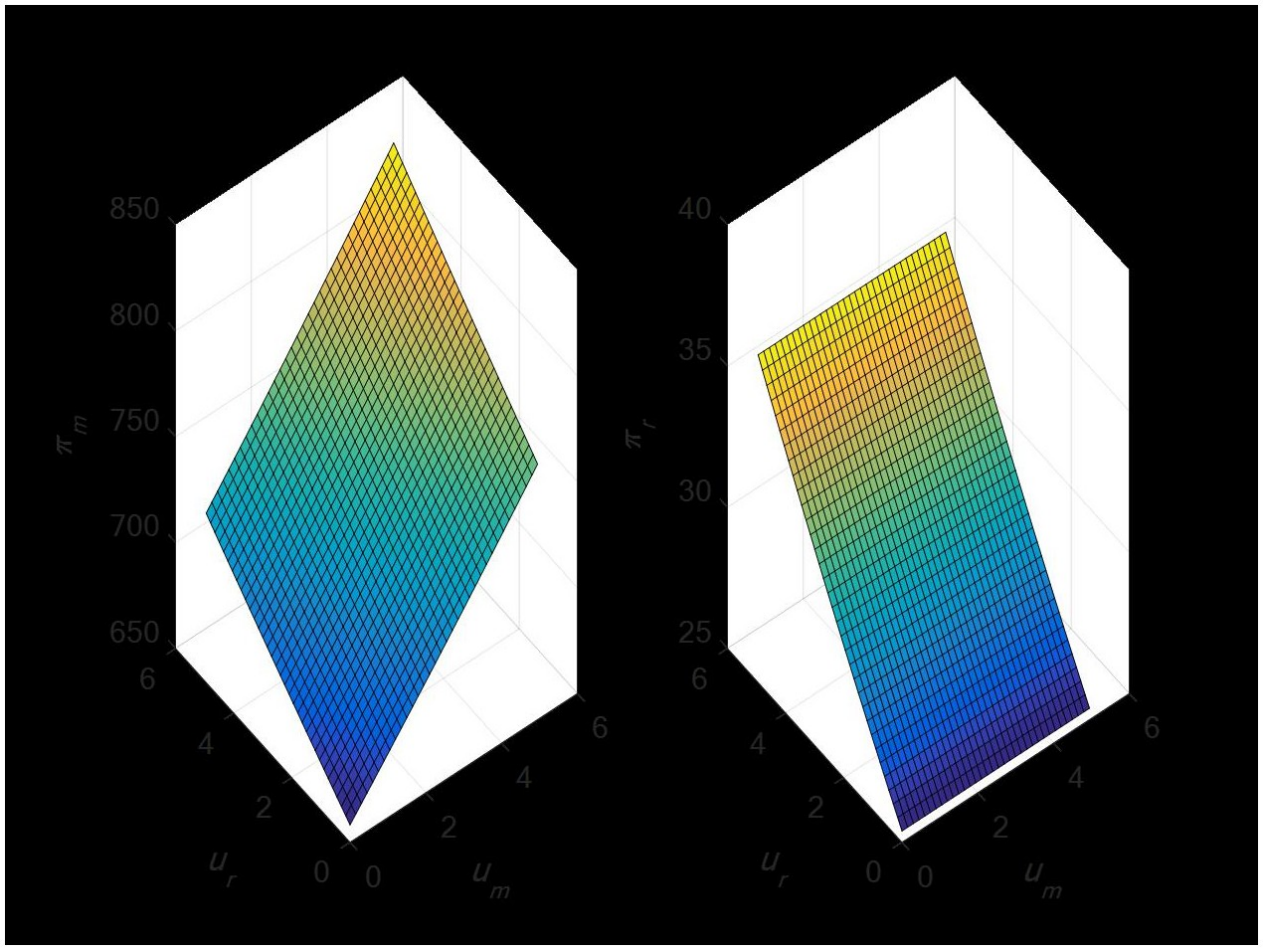
Impact of random demand on dual-channel supply chain profits.

Fig 6 illustrates that erratic demand from the direct sales channel will only benefit the manufacturer’s profit and not the retailer’s. Random demand, however, can have a favorable impact on manufacturer and retailer earnings in the retail channel. Because random demand influences demand and ultimately leads to higher profits, manufacturers and merchants will choose prices more wisely when taking it into account than if they do not.

## 5. Conclusion

This study builds a two-stage Stackelberg game model of manufacturers and retailers based on decentralized decision-making, studies the effects of import tariffs, export tax rebates, and random demand on optimal pricing, demand, and profits of supply chain members, and constructs a dual-channel supply chain of cross-border e-commerce made up of manufacturers and a retailer. The primary study findings are summed up as follows:

The study reveals the effect of import duties on manufacturers’ and retailers’ best decision-making. In an effort to lessen the impact of import tariffs, manufacturers usually cut their direct sales prices as well as their product wholesale prices. To offset the impact of import tariffs, retailers can increase retail prices. Import tariff changes have a greater influence on manufacturers’ pricing decisions compared to retailers. Tariff-induced increases in product prices result in a decline in market demand for traditional retail channels as well as online direct sales channels. The demand for direct sales channels is more affected by import taxes than the demand for retail channels. The impact of tariffs is not sufficiently offset by price increases, which results in lower earnings for producers and retailers. Because import tariffs raise the cost of consumer purchases, they are bad for retailers as well as manufacturers. As a result, lowering import duties can raise export volume and manufacturers’ and merchants’ profits.

In terms of random demand, it is disclosed how manufacturers’ and retailers’ best decision-making is affected by random demand in direct sales channels and random demand in retail channels. Prices in direct sales, wholesale, and retail will rise along with the random demand for both channels—direct sales and retail—and manufacturers’ and retailers’ earnings will rise as a result. As a result, when manufacturers and merchants take random demand into account during the decision-making process, their pricing decisions turn out to be preferable than when they don’t. This ultimately influences demand and raises profitability for merchants as well as producers.

The following are this study’s management implications: 1) As import taxes rise and result in lower demand and earnings, domestic manufacturers can strengthen their position in the market by making their products more distinctive and irreplaceable. It is recommended that domestic producers keep a careful watch on tariff and export rebate policies and promptly modify their pricing and sales tactics as necessary. Manufacturers can make better price decisions and ultimately increase profitability by taking unpredictable demand into account. 2) From the standpoint of international retailers, merchants should also be aware of tariff and export rebate rules and promptly modify prices, sales tactics, and order amounts as needed. Retailers, like manufacturers, can gain from taking random demand into account when making judgments about prices, which will enhance their profitability.

Only import tariffs, export tax rebates, and random demand are taken into account in this paper’s analysis; the manufacturer-led dual-channel pricing dispersion decisions are the primary focus. However, exchange rates and other cost considerations would affect manufacturers’ decisions during the actual cross-border export production processes. Furthermore, a cross-border supply chain typically consists of several international retailers and one domestic producer. Thus, the next stage in studying the cross-border dual-channel decision-making dilemma of one producer and many overseas merchants can take the exchange rate and other aspects into account.
